# Fertility-sparing treatment for atypical polypoid adenomyoma

**DOI:** 10.1016/j.gore.2025.101714

**Published:** 2025-03-03

**Authors:** Isabel Beshar, Susan Lang, Oliver Dorigo, Brooke E Howitt, Caroline Liu, Amer Karam

**Affiliations:** a Department of Obstetrics & Gynecology, Stanford University, 453 Quarry Road, Palo Alto, CA 94304, USA; b Division of Gynecologic Oncology, Department of Obstetrics & Gynecology, Stony Brook Medicine, Stony Brook, NY 11794, USA; c Division of Gynecology Oncology, Department of Obstetrics & Gynecology, Stanford University, 453 Quarry Road, Palo Alto, CA 94304, USA; d Department of Pathology, Stanford University, 300 Pasteur Dr Rm H2110, Palo Alto, CA 94304, USA; e Stanford School of Medicine, Stanford University, 291 Campus Drive, Stanford, CA 94305, USA

**Keywords:** Atypical polypoid adenomyoma, Hysteroscopy, Endometrial hyperplasia, Resection

## Abstract

**Introduction:**

Atypical polypoid adenomyoma (APA) has classically been described as a benign lesion of the endometrium; however, recent studies have identified risk of progression to malignant pathology. Standard treatment includes hysterectomy but since many patients with APA are young and desire fertility, uterine-sparing options have been explored. In this study, we examine long-term outcomes of fertility-sparing treatment, including hysteroscopic resection and progesterone therapy, on progression to hyperplasia or endometrial carcinoma.

**Methods:**

We performed a retrospective cohort study of patients with APA from January 1st 2000 to December 31st 2023 at our quaternary care center. Sociodemographic factors, treatment options (including hysterectomy, hysteroscopy, chemoradiation, and/or hormonal therapy), pathology pre- and post-treatment, and live birth rates, were abstracted from the record. Institutional review board approval was obtained prior to data collection.

**Results:**

Sixty-six patients were included in our study time-period. One in three patients (n = 37, 60.7 %) in our cohort opted for fertility-sparing treatment, especially among young (mean age 33.6), nulliparous patients. Most patients underwent hysteroscopic resection (70.8 %), compared to progesterone-only therapy (16.7 % with intrauterine device (IUD) and 12.5 % with oral progesterone). Over two decades, 33.3 % of our cohort progressed to hyperplasia or carcinoma; 29.2 % had persistence of APA pathology; and 33.3 % had resolution of APA. On average, patients progressed within 4.5 years of therapy. There were three births.

**Discussion:**

High rates of resolution of APA pathology were observed amongst those undergoing hysteroscopic resection with or without placement of IUD. While not statistically significant due to our sample size, lower rates of resolution were observed among those on hormonal therapy alone, especially oral progesterone.

## Highlights

• Atypical polypoid adenomyoma (APA), defined as complex glands within fibroid stroma, is a rare endometrial lesion.
• However, incidence of APA is rising, particularly amongst reproductive-aged patients.
• Fertility-sparing treatment may be a feasible option for these women.
• Hysteroscopic resection has the highest rates of regression of APA among these patients.
• Hormone-only therapy, including oral progesterone or IUD, has lower rates of regression.

## 1 Introduction

Atypical polypoid adenomyoma (APA) is a rare lesion of the endometrium. Histologically, APA is defined by architecturally complex endometrial glands in a background of fibromyomatous stroma (Skala, 2024). It was first described in the 1981 by MT Mazur, who identified five unusual polypoid lesions with smooth muscle mesenchyme in pre-menopausal women (Mazur, 1981). On ultrasound or magnetic resonance imaging (MRI), APA is indistinguishable from other intrauterine pathologies, such as polyp or fibroid and as such remains a histologic diagnosis (Biasioli et al., 2020). APA typically arises in patients of reproductive age, with many initial studies diagnosing APA during infertility workup, although it may also be found in the post-menopausal period (Wong et al., 2007, Raffone et al., 2019). APA classically affects the lower uterine segment and may cause irregular or post-menopausal bleeding in patients (Skala, 2024).

While APA is considered a “benign” lesion of the endometrium, it can co-exist with endometrial hyperplasia or endometrioid adenocarcinoma and is associated with risk of progression to hyperplasia or cancer [2. In Japan, a cohort study of 29 patients found that APA co-existed with well-differentiated endometrioid adenocarcinoma in five samples (17.2 %) [6. Initial studies found APA also has a high recurrence rate, despite transcervical resection (Young et al., 1986). A systematic review of over 63 studies identified concurrent endometrial adenocarcinoma in 4.8 % of patients, and progression to cancer in those with APA alone was 10.1 % (Mikos et al., 2019). Other studies have found similar progression rates, with estimates ranging from 8-16 % (Raffone et al., 2019, Sun et al., 2023). While the exact pathophysiology of APA remains elusive, it has been theorized that prolonged estrogenic stimulation may predispose to developing APA [10. In the mid-1990s, Fukunaga et al. found that the majority of APA stained positive for estrogen-receptor (Fukunaga et al., 1995). More recently, risk factors for unopposed estrogen exposure, such as obesity, have been implicated in the development of APA (Thanasa et al., 2023). This is supported by studies that suggest molecular and immunohistochemical staining of APA make it “most analogous to a localized form of atypical hyperplasia” (Neˇmejcova et al., 2015).

Some studies have explored fertility-sparing treatment options for patients with APA since many of them are nulliparous and desire future fertility. Sardo et al. first reported on the possibility of fertility-sparing treatment for APA in 2007, describing a case report of woman who had complete resection of APA by hysteroscopy (Di Spiezio et al., 2008). A retrospective cohort study of 25 patients found that a series of four hysteroscopic resections can be a safe approach in select candidates, with a complete response in 84 % of patients (Casadio et al., 2022). Hormonal therapy has also been postulated as a potential treatment for APA: Nomura et al. described a cohort of 18 women in Tokyo who underwent treatment with oral medroxyprogesterone acetate, with only one patient progressing to endometrioid endometrial adenocarcinoma (Nomura et al., 2018). However, given the high recurrence risk as well as progression to malignancy, close follow up and ultimate treatment with hysterectomy remain the standard of care.

Studies examining the long-term outcomes of hysteroscopic resection of APA are limited. This retrospective cohort study sought to explore treatment options for APA, particularly among patients seeking fertility or uterine-sparing options. Our primary outcome was progression of APA to hyperplasia or malignancy. Our secondary outcomes were persistence of APA lesions documented and the overall fertility rate.

## 2 Methods

### 2.1 Study Population

**Fig. 1:**
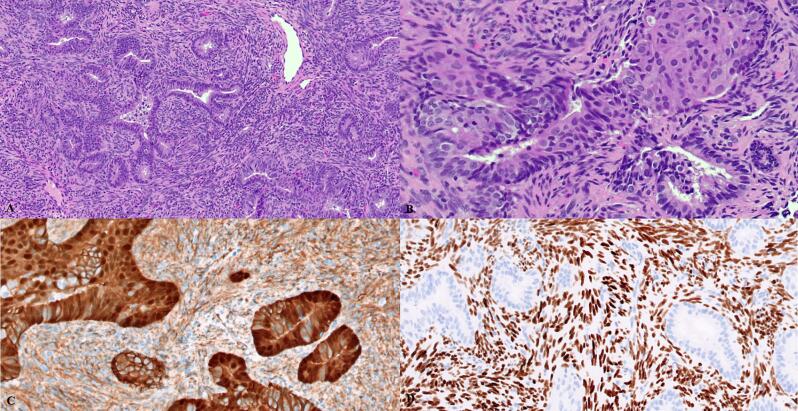
Histopathologic features of atypical polypoid adenomyoma (APA). A. Low-power magnification reveals angulated branching glands set in a fibromyomatous stroma. B. Squamous morular metaplasia is usually present at least focally and may be extensive. C. Beta-catenin is often aberrantly expressed, as shown here with nuclear positivity. D. SATB2 is often positive in the fibromyomatous stroma of APA.

Following approval from our Institutional Review Board, we performed a retrospective cohort study of patients with pathology-proven APA from January 1st 2000 to December 31st 2023 at our quaternary care center. We included any samples with APA, including if they had APA identified in a background of other concurrent pathology (such as hyperplasia or carcinoma) (example sample seen in Fig. 1**).**

### 2.2 Variables *&* Outcomes

Sociodemographic factors, including age, BMI, parity, race/ethnicity, and menopausal status were abstracted from the chart. Relevant clinical information, including diagnosis of anovulation and/or infertility were collected.

Outcomes included treatment modality, such as uterine-sparing options (hysteroscopic resection) and non-uterine sparing options (i.e., minimally invasive hysterectomy, abdominal hysterectomy), chemotherapy, radiation therapy, or hormonal therapy (i.e., progesterone intrauterine device or progesterone oral pills). Where appropriate, combinations of stated therapies above were identified. Data on progression of pathology, including endometrial hyperplasia, endometrioid endometrial carcinoma, and other histologies was collected. We noted persistence of pathology and calculated time to progression or time to resolution of lesion. Finally, among those undergoing fertility-sparing treatment, we collected the live birth rate.

### 2.3 Statistical analysis

Analyses were performed using SAS OnDemand (SAS Institute Inc, Cary, NC). Baseline characteristics in socio-demographic and reproductive health characteristics were reported as frequencies and percentages. χ^2^ tests were used to compare categorical variables and outcomes between the two groups, with Fisher exact tests for categorical analyses with sample sizes under five. In all analyses, a p-value of 0.05 or less was considered statistically significant.

## 3 Results

Sixty-six patients were included in our 23-year time period (Table 1). Five patients declined intervention and were removed from the analysis (n = 61).

**Table 1:** APA exists concurrently with hyperplasia or endometrial carcinoma in a high number of specimens.

	***Concurrent***
**Endometrial Hyperplasia**	9 (14.8 %)
**FIGO 1, 2 Endometrioid Endometrial Carcinoma**	12 (19.7%)
*FIGO Grade 1*	11
*FIGO Grade 2*	1
**Non-Endometrioid Carcinoma**	1 (1.6 %)

The mean age at diagnosis was 37.6 years [range: 32.0, 41.0 years, median age: 36.0], with a mean BMI of 32.9 kg/m^2^ [range: 25.0, 39.0 kg/m2, median BMI: 31.3]. Most patients were white (55.8 %) of non-Hispanic ethnicity (78.7). They were largely nulliparous (73.7 %), followed by those with two or more births (14.7 %). 38.2 % (n = 21) of our cohort had a diagnosis of ovulatory dysfunction or irregular cycles including 23.0 % (n = 14) of patients with the diagnosis of PCOS. Over one in three (32.8 %, n = 20) reported infertility.

Oncologic outcomes of the full cohort are reported in Table 1. At time of final diagnosis (pathology via hysteroscopy, endometrial biopsy, and/or hysterectomy), 9 (14.8 %) patients had APA concurrent with complex hyperplasia. Twelve (19.7 %) patients were found to have APA concurrent endometrioid adenocarcinoma (11 with FIGO grade 1 and one FIGO grade 2). One patient had APA concurrent with non-endometrioid histology (clear cell carcinoma).

Thirty-seven patients (60.7 %) opted for definitive therapy (Table 2**)**. In this cohort, most patients (n = 31, 83.8 %) underwent minimally invasive hysterectomy, while six patients had total abdominal hysterectomy (16.2 %). Six patients went on to receive adjuvant chemotherapy and/or external beam radiation/brachytherapy after hysterectomy.

**Table 2:** Sociodemographic and Reproductive Characteristics of the Overall Cohort, Fertility-Sparing Treatment Cohort and Definitive Treatment Cohort.

	**Overall Cohort** **(n = 61)**	**Fertility-Sparing Cohort (n = 24)**	**Definitive-Treatment Cohort (n = 37)**
**Age in years** (mean, range)	37.6 (32.0–41.0)	33.6 (29.0–36.5)	40.2 (34.0, 46.0)
**BMI kg/m2** (mean, range)	32.9 (25.0–39.0)	29.7 (23.0, 37.0)	35.1 (26.0, 43.0)
**Race** (n, %)			
*White*	36 (54.5)	11 (45.8)	23 (62.2)
*Asian*	18 (27.3)	10 (41.7)	6 (16.2)
*Black*	2 (3.0)	1 (4.2)	1 (2.7)
*Unknown*	10 (15.2)	2 (8.3)	7 (18.9)
**Ethnicity** (n, %)			
*Hispanic*	13 (19.7)	5 (20.8)	8 (21.6)
*Non-Hispanic*	53 (80.3)	19 (79.2)	29 (78.4)
**Parity** (n, %)			
*Nulliparous*	48 (71.6)	20 (83.3)	25 (67.6)
*One birth*	5 (7.5)	2 (8.3)	1 (2.7)
*Two or more births*	9 (13.4)	1 (4.2)	8 (21.6)
*Unknown*	4 (6.0)	1 (4.2)	3 (8.1)
**Diagnosis of Infertility** (n, %)	23 (38.3)	12 (50.0)	8 (21.6)
**Diagnosis of Anovulation** (n, %)	23 (34.9)	12 (54.6)	9 (27.3)

Twenty-four patients (37.3 %) opted for fertility-sparing treatment (Table 2). Overall, these patients were younger (with a mean age of 33.6 years [range: 29.0, 36.5 years], median age: 33.0) with lower BMIs (mean BMI 29.7 kg/m2 [range: 23.0, 37.0], median BMI: 28.0). They also had fewer children, with 83.3 % reporting nulliparity.

In this cohort, 17 patients (70.8 %) underwent hysteroscopic resection, while four patients (16.7 %) opted for levonorgestrel intrauterine device (IUD) and three patients (12.5 %) opted for oral progesterone therapy. Of those opting for hysteroscopic resection, five also had IUDs placed.

Overall, one third (n = 8, 33.3 %) of this cohort had progression to hyperplasia or cancer (Table 3**).** Seven patients (29.2 %) had persistence of APA despite therapy. Eight patients (33.3 %) had resolution of APA pathology confirmed on repeat pathology. One patient did not have repeat sampling.

**Table 3:** Persistence of APA or progression to pre-cancer or cancerous lesions occurred in over half of the cohort opting for fertility-sparing treatment. One patient did not undergo repeat sampling.

	**Progression to hyperplasia/cancer** **(n = 8)**	**Persistence of APA (n = 7)**	**Resolution of APA (n = 8)**
Hysteroscopic Resection (n = 12)	4 (33.3)	3 (25.0)	4 (33.3)
*With IUD Placement* (n = 5)	1 (20.0)	1 (20.0)	3 (60.0)
Intrauterine Device Placement (n = 4)	1 (25.0)	2 (50.0)	1 (25.0)
Oral Progesterone Therapy (n = 3)	2 (66.7)	1 (33.3)	0 (0)

Among those who underwent hysteroscopic resection, one third (n = 4, 33.3 %) progressed, with three patients developing complex atypical hyperplasia and one patient developing FIGO grade 1 endometrioid endometrial adenocarcinoma. In this group undergoing only hysteroscopic resection, another one third (n = 4) showed resolution of APA pathology. Three patients in this group had persistence of APA lesions. Among those undergoing combination hysteroscopy and IUD placement, three patients (66.7 %) showed resolution in APA lesions. One patient opted for hysteroscopic resection and IUD in the setting of APA in a background of some endometrioid endometrial adenocarcinoma and progressed on this therapy to FIGO grade 1 endometrioid pathology.

In those undergoing progesterone therapy alone, four patients opted for IUD placement and three opted for oral progesterone therapy. All of the patients opting for oral progesterone therapy progressed, two to FIGO grade one endometrioid endometrial adenocarcinoma and one to hyperplasia.

**Fig. 2:**
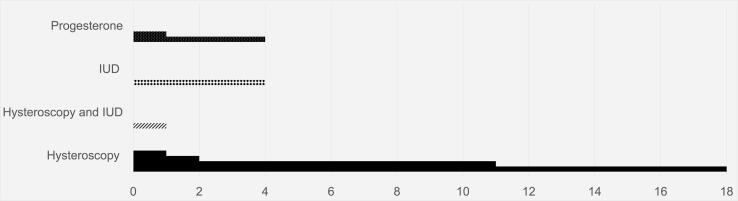
Time to progression, in months, by patient organized by treatment time. Each line represents a patient. Median time to progression was two years.

On average, patients progressed within four and a half years of therapy (range: 6 months to 18 years, median: 2 years) (Fig. 2**)**. All patients undergoing hysteroscopic resection who had progression had hysteroscopy at time of original diagnosis of APA; and a majority underwent endometrial biopsies for follow up sampling at the time of their progression. In this cohort of 24 patients who opted for fertility preservation, there were three (12.5 %) documented pregnancies.

## 4 Discussion

In our study, more than a third of our patients with APA opted for fertility-sparing treatment. In this younger, mostly nulliparous cohort, patients opted for hysteroscopy with or without levonorgestrel IUD placement, IUD placement alone, or oral progesterone therapy alone.

In our cohort, 33.3 % of patients progressed to hyperplasia or cancer and 29.2 % had persistent disease. Among those who progressed after hysteroscopic resection with or without IUD placement, three developed complex hyperplasia with atypia and two patients developed FIGO grade 1 endometrioid endometrial adenocarcinoma. Among those on progesterone therapy, two patients with IUDs progressed to FIGO grade 1 endometrioid endometrial adenocarcinoma and two patients on oral progesterone developed hyperplasia. In our overall sample, there was a high prevalence of hyperplasia and cancer at the time of APA diagnosis, with concurrent hyperplasia in 9 (14.8 %) and concurrent cancer in 15 (24.5 %) patients at time of diagnosis. This aligns with prior research suggesting APA may exist with areas of “focal canceration” in the endometrium (Sun et al., 2023).

Comparison of fertility sparing treatment modalities is difficult given our sample size. We did observe high rates of resolution amongst those undergoing hysteroscopic resection with placement of IUD and high rates of progression to hyperplasia or carcinoma among those on hormonal therapy alone. Our findings were not statistically significant, but corroborate the existing, if limited, research on fertility-sparing treatment for APA, which has shown higher response rates with hysteroscopic resection than progesterone therapy alone (Raffone et al., 2019, Casadio et al., 2022) and may parallel existing data showing superiority of resection and progesterone therapy in complex endometrial hyperplasia than progesterone therapy alone (Giampaolino et al., 2019). Indeed, APA is a discrete, circumscribed mass with prominent and dense fibromyomatous stroma; thus it is logical that APA may be more responsive to resection than changes in the hormonal milieu (Casadio et al., 2022).

Our findings support the recently published study by Sun et al. examining 203 patients with APA (Sun et al., 2023). They found that patients undergoing hysteroscopic resection had a 20 % recurrence rate and a 9.1 % chance of progressing to endometrial cancer. In this study, a four-step treatment method for hysteroscopy was utilized, as described by Di Spiezio Sardo et al (resection of the APA lesion, removal of the endometrium adjacent to the lesion, removal of the superficial myometrial tissue at the lesion, and random-spot biopsies in other parts) (Sun et al., 2023). Wang et al. also reviewed oncologic and fertility outcomes of 86 patients with APA, finding that high-dose progestin treatment after hysteroscopic resection may delay or event prevent recurrence of disease (Wang et al., 2024). Among their cohort of 62 patients opting for fertility-sparing or conservative treatment, 17 patients had no recurrence with high-dose progestin treatment with Megace (typically 160 mg daily) following hysteroscopic resection (Wang et al., 2024). They also identified a high co-occurrence rate between patients with APA and hyperplasia and endometrioid endometrial adenocarcinoma, with as many as 56.9 % of their patients harboring both pathologies.

Finally, over one third of our patients had anovulatory cycles and nearly a quarter (24 % of the overall cohort) had an official diagnosis of PCOS which is higher than expected. While APA has previously been theorized to occur in a background of unopposed estrogen, to date little data has linked APA to PCOS (Fukami et al., 2016, Butureanu et al., 2023). PCOS results in irregular cycles and anovulation, leading to unstable endometrial linings and increased rates of endometrial hyperplasia and carcinoma; our data suggests this pathophysiology may be associated with an increased prevalence of APA lesions. Since long-term epidemiology data suggests PCOS is underdiagnosed by as much as 50 %, physicians should have a low threshold to perform endometrial sampling in patients with cycle derangements or hyperandrogenic symptoms (The Lancet Regional Health, 2022).

The strengths of this study include our large cohort size and prolonged study interval, as we identified 61 specimens across two decades of data collection at our institution. We are limited by the retrospective nature of this study with heterogeneous treatment plans, which limits our ability to make predictive conclusions about optimal management of APA. There was also variety in sampling methods (hysteroscopy versus endometrial biopsy) of these patients, which may have impacted the accuracy of the initial diagnosis.

Our study demonstrates that many patients are interested in fertility-sparing approaches to treating APA, especially among young, nulliparous patients. Higher rates of regression may be seen in those undergoing hysteroscopic resection of the lesion. Further research could explore combination efforts for fertility-sparing options for APA as well as genetic germline testing of patients with APA.

**Funding Statement**

This research did not receive any specific grant from funding agencies in the public, commercial, or not-for-profit sectors.

**Data Sharing Statement**

Raw data and data dictionary may be made available upon request.

**Prior presentation**

Presented at the annual meeting of the Western Association of Gynecologic Oncology (June 2024, Seattle) and at the Society for Gynecology Oncology (March 2025, Seattle).

**Contribution Statement**

Karam and Lang conceptualized this study. Beshar and Liu conducted data collection and Beshar contributed statistical analysis and review. Beshar drafted the initial manuscript. Lang, Karam, Dorigo and Howitt contributed to figures and data curation as well as draft review. Karam contributed project supervision and administration.

## CRediT authorship contribution statement

**Isabel Beshar:** Writing – original draft, Project administration, Methodology, Formal analysis, Data curation. **Susan Lang:** Writing – review & editing, Supervision, Methodology, Conceptualization. **Oliver Dorigo:** Writing – review & editing, Validation. **Brooke E Howitt:** Writing – review & editing, Visualization, Data curation. **Caroline Liu:** Data curation. **Amer Karam:** Writing – review & editing, Supervision, Project administration, Methodology, Investigation, Conceptualization.

## Declaration of competing interest

The authors declare that they have no known competing financial interests or personal relationships that could have appeared to influence the work reported in this paper.
